# An open-label, dose-finding study of the combination of satraplatin and gemcitabine in patients with advanced solid tumors

**DOI:** 10.3389/fonc.2012.00175

**Published:** 2012-11-22

**Authors:** Eugenio Donato Di Paola, Silvia Alonso, Rosa Giuliani, Fabio Calabrò, Antonietta D’Alessio, Giovanni Regine, Linda Cerbone, Laura Bianchi, Andrea Mancuso, Sabine Sperka, Marcel Rozencweig, Cora N. Sternberg

**Affiliations:** ^1^Department of Medical Oncology, San Camillo and Forlanini HospitalsRome, Italy; ^2^Department of Science of Health, School of Medicine, University “Magna Graecia”Catanzaro, Italy; ^3^Department of Radiology, San Camillo and Forlanini Hospitals RomeItaly; ^4^Clinical Operations, Agennix AGHeidelberg, Germany; ^5^GPC Biotech Inc., PrincetonNJ, USA

**Keywords:** satraplatin, oral platinum, phase I study, prostate cancer, solid tumors, chemotherapy

## Abstract

**Purpose:** Satraplatin is a third generation oral platinum, which has demonstrated antitumor activity. The aim of this phase I study was to determine the maximum tolerated dose (MTD) of the combination of satraplatin and gemcitabine in patients previously treated with chemotherapy and in patients without prior chemotherapy. **Patients and Methods:** Two separate MTDs were planned in two different patient groups (those with and without prior chemotherapy treatment). Dose escalations were planned in cohorts of three patients. Tumor measurements were obtained every two cycles. Assessment of response was performed according to Response Evaluation Criteria in Solid Tumors (RECIST criteria v.1.0). **Results:** Thirty subjects were enrolled. A MTD of gemcitabine 1000 mg/m^2^ days 1 and 8 plus satraplatin 60 mg/m^2^ days 1–3, every 21 days was determined in the prior chemotherapy group. No MTD could be determined for the no prior chemotherapy group treated with this schedule. Five patients completed 12 treatment cycles; 22 serious adverse events (SAE) were observed. Although not an entry criteria, overall confirmed response was observed in 17 (24%) evaluable patients (complete response, CR = 1 and partial response, PR = 3) and in 3/7 (43%) patients with measure prostate cancer lesions. **Conclusions:** In this phase Ib study, the combination of satraplatin and gemcitabine demonstrated to be safe and efficacious in particular in patients with prostate cancer.

## INTRODUCTION

Satraplatin is a third generation oral platinum complex that has demonstrated activity against several platinum-sensitive and -resistant human tumor cell lines (Twentyman et al., 1992; Kelland et al., 1993; Mellish et al., 1993; Orr et al., 1994; Raynaud et al., 1996).

Preclinical and clinical studies have shown that satraplatin can potentiate the effects of radiotherapy (Van de Vaart et al., 1997; Amorino et al., 1999, 2000; George et al., 2001). In clinical studies, the most frequent dose limiting toxicity (DLT) observed with satraplatin was myelosuppression (leucopenia and thrombocytopenia; McKeage et al., 1995, 1997; Beale et al., 1998; Fokkema et al., 1999; Kurata et al., 2000; George et al., 2001). Of importance, no significant nephrotoxicity or neurotoxicity was reported with satraplatin in either preclinical or clinical studies (McKeage et al., 1993, 1994, 1995, 1997; Beale et al., 1998; Sessa et al., 1998; Fokkema et al., 2000; Vouillamoz-Lorenz et al., 2003; Ricart et al., 2009; Galsky et al., 2012).

In phase II trials, single-agent satraplatin demonstrated activity in patients with small cell lung cancer (SCLC), relapsed ovarian cancer, and prostate cancer (Judson et al., 1997).

In a phase II trial of satraplatin in 39 chemo-naive patients with progressive castration-resistant prostate cancer (CRPC), 7 of 22 (32%) patients had a PSA response, toxicity was mainly hematologic, with grade 3/4 non-hematologic toxicities including transient increases in aspartate transaminase and bilirubin (Latif et al., 2005). These results led the European Organization for Cancer Research (EORTC) to initiate a phase III trial of satraplatin plus prednisone vs prednisone alone for first-line treatment of patients with CRPC (Sternberg et al., 2005). Although the target accrual was 380 patients, only 50 patients were enrolled when the study was terminated early due to a company decision. This trial demonstrated that the combination of satraplatin and prednisone resulted in a significant increase in PSA response compared to prednisone alone (33 vs 9%; *P* = 0.046), and improvement in progression-free survival (PFS; 5.2 vs 2.5 months; *P* = 0.023; Sternberg et al., 2005).

Encouraging results of this EORTC trial led to development of the SPARC (Satraplatin and Prednisone against Refractory Cancer) study (Sternberg et al., 2009). This trial was a phase III randomized double-blind study in which satraplatin plus prednisone was compared to placebo plus prednisone as second-line treatment in patients with CRPC who had received one prior line of cytotoxic chemotherapy. The PFS was 11.1 weeks on the satraplatin and prednisone arm and 9.7 weeks on the placebo and prednisone arm (*P* < 0.001). The median time to pain progression was 66.1 weeks for satraplatin and 22.3 weeks for placebo. A PSA response was observed in 25.4 and 12.4% of patients, respectively (*P* < 0.001). Despite the improvement in PFS and the palliative effects in favor of satraplatin, overall survival (14.3 months in both arms), the preferred endpoint for regulatory approval by the FDA, for prostate cancer clinical trials as most patients have inevaluable bone disease, was not extended by satraplatin.

Gemcitabine is frequently used in the treatment of several tumor types including: breast, bladder, non-SCLC, and pancreatic cancers. Activity has also been reported in biliary tract, cervical, gall bladder, and ovarian cancers. Gemcitabine has been combined with several different chemotherapeutic agents and has shown activity with platinums, taxanes, anthracyclines, 5-fluorouracil, irinotecan, vinorelbine, and others (Pollera et al., 1994; Glimelius et al., 1996; Burris et al., 1997; Sternberg, 2000).

The rationale for this study was based primarily upon the general synergy between platinum compounds and gemcitabine. Simultaneous drug combination of satraplatin and gemcitabine in UM-UC-3 cells was often more effective than the individual drug treatments but overall showed less than additive effects (GPC Biotech AG, 2005). In this dose finding study the maximum tolerated dose (MTD) of the combination of satraplatin and gemcitabine was evaluated. The hope was to subsequently explore the combination in a variety of solid tumors.

## PATIENTS AND METHODS

### PATIENT SELECTION CRITERIA

Eligibility criteria included histological diagnosis of metastatic or advanced-stage malignant solid tumors that had progressed following standard therapy or in whom no standard effective treatment was available. Subjects may have received up to two prior lines of chemotherapy for their metastatic disease. Patients with prior therapy with a platinum agent or gemcitabine were allowed as long as they obtained objective response to one of these agents and their relapse occurred after 6 months. Other criteria included: age ≥18 years, ECOG performance status (PS) 0–2, adequate bone marrow function, adequate renal and hepatic function, measurable or non-measurable disease according to Response Evaluation Criteria in Solid Tumors (RECIST criteria v.1.0).

An independent ethical committee at San Camillo and Forlanini Hospitals approved the protocol. All patients signed written informed consent according to ICH Good Clinical Practice prior to study entry. The trial was conducted according to the Declaration of Helsinki and its amendments.

### TRIAL DESIGN AND PROCEDURES

The study was conducted as a single-center, open label, dose escalation study combining gemcitabine with satraplatin in subjects with advanced solid tumors. Patients were stratified into two groups at registration according to the number of lines of prior chemotherapy (0 vs 1–2). Two separate MTDs were planned for patients with and without previous chemotherapy. The recommended phase II dose was defined as the same dose level as the MTD.

Initially, gemcitabine was given by IV infusion on days 1, 8, and 15 every 28 days followed by satraplatin p.o. for the first 5 days of each cycle (every 28-day schedule, part 1). Seventeen patients were enrolled and treated according to this 28-day schedule. Due to the increasingly common practice of giving gemcitabine and cisplatin on an every 3-week schedule and thrombocytopenia encountered with gemcitabine on the 28-day schedule, the protocol was subsequently amended to an every 21-day schedule (part 2). The second part of the study included 13 additional patients.

At screening and prior to each gemcitabine infusion, medical history, concomitant medication and PS were recorded. Adverse events were monitored throughout the trial. In both schedules patients were to receive up to 12 cycles. Treatment was discontinued after a dose delay of more than 3 weeks, need for more than one dose reduction, creatinine clearance below 40 ml/min, documented disease progression, or initiation of confounding anti-cancer therapy.

### DOSE ESCALATION

The starting dose and dose escalation of gemcitabine and satraplatin in patients with prior chemotherapy and patients with no prior chemotherapy in parts 1 and 2 of the study are summarized in **Tables 1 and 2**.

**Table 1:** Dose escalation in part 1 study (every 28-day schedule).

Dose level	Gemcitabine mg/m^2^ days 1, 8, and 15^*^	Patients with prior chemotherapy	Patients with no prior chemotherapy
		Satraplatin mg/m^2^/day given daily for 5 days	Satraplatin mg/m^2^/day given daily for 5 days
Starting	800	40	60
+1	1000	40	60
+2	1000	60	80
+3	1000	80	100

* Cycles are to be repeated every 28 days.

**Table 2:** Dose escalation in part 2 study (every 21-day schedule).

Dose level	Gemcitabine mg/m^2^ days 1 and 8^*^	Patients with prior chemotherapy	Patients with no prior chemotherapy
		Satraplatin mg/m^2^/day given daily for 3 days	Satraplatin mg/m^2^/day given daily for 3 days
Starting	1000	60	80
+1	1000	80	100
+2	1000	100	120
+3	1250	100	120

* Cycles are to be repeated every 21 days.

Dose escalations were planned in cohorts of three patients. If none of the three patients experienced a DLT during the first cycle, the next three patients were treated at a higher dose level. If any of the three patients experienced a DLT, three additional patients were treated at the same dose. If any of the six patients experienced a DLT, the MTD was exceeded and three additional patients had to be treated at the lower dose (if only three patients were previously treated at that dose). The MTD was defined as the dose level at which 0/6 or 1/6 patients experience DLTs with the next higher dose having at least 2/3 or 2/6 patients experiencing a DLT.

Toxicities were graded on the basis of Common Terminology Criteria for Adverse Events (CTCAE) version 3.0. A DLT was defined as a drug-related CTCAE G3 or G4 non-hematologic toxicity (except reversible emesis or diarrhea) or G4 neutropenia of more than 7 days and/or complicated by infection or G3–G4 thrombocytopenia or any bleeding episode requiring platelet transfusion, or delayed recovery (to G1 or baseline, except alopecia). A toxicity related to the combination treatment which delayed initiation of the next cycle by more than 2 weeks was also a DLT.

### SAFETY ASSESSMENT

Safety was assessed weekly by physical examination, vital signs and laboratory measurements. Subjects were followed for adverse events for at least 30 days after the last dose of therapy. All study-related adverse events were followed to resolution or stabilization.

### TUMOR ASSESSMENTS

Subjects with measurable disease who had completed at least two cycles of study treatment and had at least one disease assessment following the initiation of study treatment were considered evaluable for disease response. Tumor measurements were obtained every two cycles of treatment. Response was assessed according to RECIST criteria v.1.0 (Therasse et al., 2000). Subjects who discontinued for toxicity or after completion of 12 cycles without disease progression were followed every 3 months up to 1 year or until progressive disease, subsequent anti-tumor treatment, or death.

### STATISTICAL METHODS

The statistical analysis was only descriptive.

## RESULTS

### PATIENT CHARACTERISTICS

Thirty patients were entered onto the study. Of these, 17 were in part 1 (every 28-day schedule) and 13 in part 2 (every 21-day schedule). Patient characteristics of both groups are described in **Table 3**.

**Table 3:** Patients characteristic.

Characteristics	All patients	Part 1		Part 2	
		No previous chemotherapy	Previous chemotherapy	No previous chemotherapy	Previous chemotherapy	
*N*. of patients	30	11	6	2	11
**Gender**
Male	25	8	5	2	10
Female	5	3	1	0	1
**Age (year)**
Median	68	68	63	61	67
Range	36–81	40–81	36–74	49–73	59–77
**PS**
0	8	2	2	1	3
1	19	8	3	0	8
2	3	1	1	1	0
***N*. of previous chemotherapy regimens**
0	13	11	0	2	0
1	11	0	4	0	7
2	6	0	2	0	4
**Time since diagnosis (months)**
Median	16.7	3.7	26.9	8	78.5
Range	0.1–133.7	0.2–45.4	7.9–107.9	0.1–15.8	12.1–133.8
**Tumor type**
Prostate	13	0	3	0	10
Pancreatic	6	4	0	1	1
Hepatocellular carcinoma	4	3	1	0	0
Gastric cancer	2	1	0	1	0
Papillary renal carcinoma	1	1	0	0	0
Unknown primary site	1	1	0	0	0
Biliary tract	1	1	0	0	0
Thymic	1	0	1	0	0
Bladder	1	0	1	0	0
**Target lesions**
Yes/no	23/7	9/2	5/1	2/0	7/4
Median	2	3	2	3	1
Range	0–8	0–6	0–8	1–5	0–5

### TREATMENT, MTD, AND DLTs

The median number of cycles administered was 3. Five patients completed a total of 12 treatment courses, the maximum number of cycles per protocol. Details are reported in **Table 4**.

**Table 4:** Treatment (cycles administered).

*N*. of cycles administered	All patients	Part 1		Part 2	
		No previous chemotherapy	Previous chemotherapy	No previous chemotherapy	Previous chemotherapy
Total	137	38	25	4	70
Median	3	2	2	2	6
Range	1–12	1–12	2–12	1–3	2–12
12 cycles	5	1	1	0	3

#### Every 28-day schedule part I of the trial

The trial was initiated with a 28-day schedule. The starting dose of satraplatin was 40 mg/m^2^ in pretreated and 60 mg/m^2^ in chemo-naive patients (equivalent to 40 and 60% of the MTD of single-agent satraplatin).

In the “previous chemotherapy” group, three patients were treated at the starting dose (gemcitabine 800 mg/m^2^ days 1, 8, and 15 plus satraplatin 40 mg/m^2^ days 1–5; every 28 days). A DLT (G3 transaminases) was observed and the cohort was expanded to three more patients. Another DLT (G3 transaminases) was observed in this cohort. Thus, no patients were treated at the next dose level. Since two DLTs occurred, no MTD could be determined.

In the “no previous chemotherapy” group, eight patients were treated at the starting dose (gemcitabine 800 mg/m^2^ days 1, 8, and 15 plus satraplatin 60 mg/m^2^ days 1–5; every 28 days). Two patients were not evaluable for the MTD and were replaced. Both patients did not receive treatment on days 8 and 15 of cycle 1, due to G3 thrombocytopenia or withdrawal of consent during cycle 1. One DLT (G3 diarrhea) was observed in one of the six evaluable patients. Therefore, three additional patients received +1 dose level (gemcitabine 1000 mg/m^2^ days 1, 8, and 15 plus satraplatin 60 mg/m^2^ days 1–5; every 28 days). At dose level +1, one DLT (G3 thrombocytopenia) was observed in one of three patients. The starting dose (level 0) was therefore determined as the MTD.

In the “previous chemotherapy” group, no MTD was determined, and in the “no previous chemotherapy” group, the MTD was determined at dose level 0. Only 6 of 17 patients received gemcitabine on D8 and D15. Therefore, the protocol was amended in both groups to a more convenient every 3-week schedule.

#### Every 21-day schedule part II of the trial

In the “previous chemotherapy” group, seven patients were treated at the starting dose (gemcitabine 1000 mg/m^2^ days 1 and 8 plus satraplatin 60 mg/m^2^ days 1–3; every 21 days). One patient was not evaluable for the MTD, as he received no treatment on day 8 due to urinary infection. One DLT (G3 thrombocytopenia) was observed in one of six evaluable patients.

In the next three patients, the dose was escalated to dose level +1 (gemcitabine 1000 mg/m^2^ days 1 and 8 plus satraplatin 80 mg/m^2^ days 1–3; every 21 days). Two DLTs were observed (G3 thrombocytopenia).

The starting dose (level 0) was, therefore, determined as the MTD and further enrolment of six patients was planned. This was stopped after the first patient exhibited a DLT (G3 thrombocytopenia) due to a decision of the sponsor.

In the “no previous chemotherapy” group, two patients were treated at the starting dose (gemcitabine 1000 mg/m^2^ days 1 and 8 plus satraplatin 80 mg/m^2^ days 1–3; every 21 days). In each patient, one DLT was observed (G3 neutropenia and G3 thrombocytopenia). Dose level 0 was closed and the MTD could not be determined.

#### Safety results

All 30 patients received at least one dose of study treatment. A total of 137 cycles were evaluated for safety and 310 non-serious adverse drug reactions (ADRs) were recorded. Eighteen patients experienced 30 SAEs including 22 that were considered “possibly related” to study treatment. The reported serious ADRs were: diarrhea (1), rectal hemorrhage (1), ALT increase (3), low hemoglobin (4), low platelets (8), neutropenia (2), elevated bilirubin (1), and deep venous thrombosis (1). G4 toxicities occurred in seven patients (three neutropenia, two anemia, one thrombocytopenia, and one pain). The predominant adverse event that occurred in greater than 20% of the patients are reported in **Table 5**. No major differences in adverse events G3–G4 were observed between the subjects in the two groups with and without prior chemotherapy.

**Table 5:** Adverse events in ≥20% of patients.

Adverse events	*N*. of patients with G1	*N*. of patents with G2	*N*. of patents with G3	*N*. of patents with G4	% of patients with (G1–G4)
Asthenia	6	12	4	0	73
Nausea	8	7	1	0	53
Thrombocytopenia	3	4	7	1	50
Neutropenia	1	3	7	3	47
Anorexia	5	8	1	0	47
Anemia	0	8	2	2	40
Fever	7	4	0	0	37
Diarrhea	3	4	2	0	30
Constipation	9	0	0	0	30
Pain	5	1	0	1	23
Edema	5	1	0	0	20

### ANTITUMOR ACTIVITY

Seventeen of the 30 patients were evaluable for objective tumor response. Thirteen patients were excluded from the analysis as per protocol because seven had no measurable disease (only non-target lesions) at inclusion, four received less than two cycles of study treatment and two patients had no further disease assessments following initiation of the study treatment.

One patient with pancreatic cancer and one target lesion in the liver showed a complete response (CR) at cycle 2 which was confirmed at cycle 4. Time to progression was 422 days. Three patients had confirmed partial response (PR). Seven had stable disease (SD) in whom two had a PR that was not confirmed. Six patients had progression disease (PD). All three patients who obtained a PR had metastatic CRPC. Two patients had target lesions in lymph nodes and in one the liver. Of the two patients that had an unconfirmed PR, one had metastatic CRPC with target lesions in lymph nodes and the other had a biliary tract tumor with four target lesions (two in the liver and two in lymph nodes). The overall response rate (RR; CR + PR) in all (4/17) evaluable patients was 24%.

## DISCUSSION

The purpose of this study was to determine the MTD and DLT of gemcitabine in combination with satraplatin in two groups of patients with advanced solid tumors. Since there was strong evidence of synergism of the combination of the two drugs in preclinical studies, it was decided to conduct the study in two groups of patients simultaneously.

The first group included patients previously treated with one or two lines of chemotherapy and for whom no further standard treatment was available. The second group included patients not previously treated with chemotherapy and in whom there was no standard treatment. This group included patients with pancreatic, gastric, hepatocellular carcinoma, biliary tract, or papillary renal cell cancers (**Table 3**).According to the protocol, more patients on the 28-day schedule at dose level 0 should have been included in order to verify if this was the recommended dose for phase II trials. However, taking into account the encouraging evidence of activity of the combination of the two drugs, as evidenced by a CR in a patient with pancreatic cancer, one PR in a patient with CRPC and one unconfirmed PR in a patient with biliary tract cancer, and the fact that only 6 of 17 patients had received gemcitabine on days 8 and 15, the protocol was amended in both groups to a more feasible schedule (gemcitabine days 1 and 8, plus satraplatin days 1–3; every 21 days).

Unfortunately, the second part of the amended study was stopped after the FDA ODAC meeting in which satraplatin and prednisone in the SPARC trial failed to reveal an OS advantage in patients with CRPC and one prior line of chemotherapy. At that time only 50% of patients in the SPARC trial had received prior first-line treatment with docetaxel which was becoming the standard first-line chemotherapy.

The main objective of the study was to explore the safety of the combination of gemcitabine with satraplatin. For this reason, measurable disease was not required in the study. Of particular interest was the activity in patients with metastatic CRPC. The cycle length of 21 days would have provided a practical schedule. In addition, the efficacy of platinums in prostate cancer has been noted. An oral platinum compound would have potentially been a very efficacious alternative to other chemotherapeutic agents (Oh et al., 2007). There were 13 CRPC patients enrolled, all of them were included after treatment with docetaxel and prednisone, and four also received a prior second line of chemotherapy.

Only seven CRPC patients had target lesions and were evaluable according to the protocol, while six were not evaluable for objective response. Four were not evaluable due to lack of measurable target lesions, one received less than two cycles and one was not re-evaluated. In this subgroup of evaluable patients with measurable lesions, the objective RR was 43%. Although the number of evaluable CRPC patients was limited, the data are reinforced by the fact that PSA responses >50% according to Prostate Specific Antigen Working Group (PSAWG) criteria were also observed in 4/12 (33%) patients.

It is unfortunate that development of satraplatin was halted as it showed potentially interesting results in this study and other studies in ovarian cancer, lung cancer and as a radioenhancer for external beam radiation therapy. In actuality, satraplatin is under development as a radioenhancer and translational research studies with biomarkers are supported through a Material Cooperative Research and Development Agreement (MCRADA) with the NIH.

## CONCLUSION

The arsenal of novel hormonal treatment for patients with CRPC has radically changed in recent years. Nonetheless, the results of this trial showed that the combination of gemcitabine and satraplatin, in particular given in an every 3 week cycle is feasible and has potential anti-tumor activity.

The results were encouraging, in particular, as second and third line chemotherapy in patients with CRPC, supported by the high objective RR of 43%. While the drug combination may be of interest in CRPC, the study did not prospectively enrolled a defined cohort of CRPC patients. Although the number of evaluable patients was limited due to study closure, the combination was feasible and patients were able to receive the maximum number of cycles permitted by the protocol (12 cycles).

In conclusion, the results of this study and the compliance with an oral platinum agent support the further development of satraplatin to better quantify its activity and safety in patients with CRPC after receiving docetaxel and other novel therapies such as abiraterone or enzalutamide. The combination was also interesting in pancreatic and biliary tumors.

## Conflict of Interest Statement

Marcel Rozencweig was consultant for and Sabine Sperka was employed at GPC Biotech Inc. The other authors declare that the research was conducted in the absence of any commercial or financial relationships that could be construed as a potential conflict of interest.
